# Prevention of Huntington's Disease-Like Behavioral Deficits in R6/1 Mouse by Tolfenamic Acid Is Associated with Decreases in Mutant Huntingtin and Oxidative Stress

**DOI:** 10.1155/2019/4032428

**Published:** 2019-03-26

**Authors:** Peng Liu, Yinjie Li, Wei Yang, Danyang Liu, Xuefei Ji, Tianyan Chi, Zhutao Guo, Lin Li, Libo Zou

**Affiliations:** ^1^Department of Pharmacology, Shenyang Pharmaceutical University, Shenyang 110016, China; ^2^Department of Clinical Pharmacy, Shenyang Pharmaceutical University, Shenyang 110016, China; ^3^Key Laboratory of Neurodegenerative Diseases (Capital Medical University), Ministry of Education, Beijing 100053, China

## Abstract

Tolfenamic acid is a nonsteroidal anti-inflammatory drug with neuroprotective properties, and it alleviates learning and memory deficits in the APP transgenic mouse model of Alzheimer's disease. However, whether tolfenamic acid can prevent motor and memory dysfunction in transgenic animal models of Huntington's disease (HD) remains unclear. To this end, tolfenamic acid was orally administered to transgenic R6/1 mice from 10 to 20 weeks of age, followed by several behavioral tests to evaluate motor and memory function. Tolfenamic acid improved motor coordination in R6/1 mice as tested by rotarod, grip strength, and locomotor behavior tests and attenuated memory dysfunction as analyzed using the novel object recognition test and passive avoidance test. Tolfenamic acid decreased the expression of mutant huntingtin in the striatum of 20-week-old R6/1 mice by inhibiting specificity protein 1 expression and enhancing autophagic function. Furthermore, tolfenamic acid exhibited antioxidant effects in both R6/1 mice and PC12 cell models. Collectively, these results suggest that tolfenamic acid has a good therapeutic effect on R6/1 mice, and may be a potentially useful agent in the treatment of HD.

## 1. Introduction

Huntington's disease (HD) is an autosomal-dominant neurodegenerative disorder, the clinical hallmarks of which include motor dysfunction, psychiatric disturbance, and cognitive deficits. HD is caused by abnormal expansion of the cytosine-adenine-guanine repeat in the *IT15* gene located on chromosome 4, resulting in the formation of a polyglutamine stretch in the N-terminus region of the Huntingtin protein (Htt) [1]. Mutant Htt (mHtt) causes selective neuronal loss in the brain. Mouse models of HD, most commonly the R6 transgenic model that expresses a truncated form of human Htt, have been primarily used to examine several therapeutic strategies [2]. Specificity protein 1 (Sp1) is a transcription factor, the target genes of which include amyloid *β* precursor protein (*APP*), *BACE1*, *Tau*, and *Htt*, which all play vital roles in neurodegenerative diseases. Because Sp1 promotes human *Htt* gene transcription [3–5], we hypothesized that the downregulation of Sp1-mediated Htt transcription may alleviate the pathogenesis of HD.

Tolfenamic acid (TA) is a nonsteroidal anti-inflammatory drug (NSAID) that decreases the expression and activity of SP1 [6]. Previous studies have reported that tolfenamic acid alleviated cognitive deficits and downregulated the expression of *BACE1*, *APP*, and phosphorylated tau in APP transgenic mice [7, 8]. Sankpal et al. reported that repeated administration of tolfenamic acid in mice did not decrease body weight nor did it exhibit a toxic effect on several organs [9], which suggests that tolfenamic acid is safe for oral administration. However, whether tolfenamic acid can prevent HD-like symptoms remains unclear.

In the present study, we investigated the effect of tolfenamic acid on R6/1 transgenic mice. First, we assessed motor function (rotarod test, grip strength test, and locomotor behavior test), memory function (novel object recognition test, Y maze test, and passive avoidance test), body weight, and brain weight. Subsequently, we tested the effect of tolfenamic acid on huntingtin levels. To further understand the molecular mechanism of action of tolfenamic acid, we tested the effect of tolfenamic acid on autophagy and oxidative stress in the brains of R6/1 transgenic mice.

## 2. Materials and Methods

### 2.1. Materials

Tolfenamic acid (purity ≥98%) was purchased from a commercial supplier (Abcam, Cambridge, MA, USA), and 3-nitropropionic acid (3-NP) was purchased from Sigma-Aldrich (MO, USA). The mouse monoclonal antibody against huntingtin (EM48) was purchased from Millipore (CA, USA). The rabbit polyclonal antibodies against LC3, P62, and HO1; the mouse monoclonal antibody against *β*-actin; and horseradish peroxidase-conjugated secondary antibodies were purchased from Proteintech (Wuhan, China). ML385, a specific Nrf2 inhibitor, was purchased from MedChem Express (NJ, USA). Nrf2 small interfering RNA (siRNA) and rabbit polyclonal antibody against Sp1 were purchased from Santa Cruz Biotechnology Inc. (CA, USA). The rabbit polyclonal antibody against NQO1 and D,L-buthionine-(S,R)-sulfoximine (BSO), a specific glutathione synthase inhibitor, was purchased from Abcam (Cambridge, MA, USA).

### 2.2. Animals

B6.Cg-Tg(HDexon1)61Gpb/JNju mice (i.e., R6/1) were procured from Nanjing Biomedical Research Institution of Nanjing University (Nanjing, China), and wild type (WT) mice were used as control. The animals were housed in polyacrylic cages (30.0 cm length × 12.0 cm height × 18.0 cm width) under standard conditions with a 12 h light/dark cycle and had *ad libitum* access to food and water. Body weight was recorded every 6-7 days from 8 weeks of age until the animals were euthanized. All procedures involving animals were performed in strict accordance with the P.R. China legislation on the use and care of laboratory animals and the guidelines established by the Institute for Experimental Animals at Shenyang Pharmaceutical University (permit number: SYPU-IACUC-C2016-2-25-183).

### 2.3. Drug and Treatment Schedule

The mice were divided into four groups of 8–10 animals each: control (WT mice); R6/1 mice (model group); R6/1 mice treated with tolfenamic acid (25 mg/kg); and R6/1 mice treated with tolfenamic acid (50 mg/kg). Ten-week-old mice were orally administered tolfenamic acid or vehicle by gavage. After the behavioral test, the mice were decapitated under ether anesthesia, and brain tissue was extracted and dissected. The right half of the brain was used for immunohistochemical staining, while striatum from the left half was used for western blotting analysis. The left half of the brain (except striatum) was used to assess oxidative stress levels. The selection of two tolfenamic acid doses was based on the study by Adwan et al. [8] and the authors' previous study.

### 2.4. Locomotor Behavior Test

Locomotor activity was assessed using a computer system and video camera when the mice were 20 weeks old. Mice were placed individually in a white PVC-enclosed chamber (25 cm long × 25 cm wide × 30 cm high) for 3 min to acclimatize to the unfamiliar environment, followed by recording of motor activity for 5 min. Exploration distance, time, and number were recorded. After each test, the floor was cleaned using ethanol (10%) to eliminate olfactory cues.

### 2.5. Grip Strength Test

The forelimb strength test was performed in mice 8 to 20 weeks old. Mice were grasped by their back and drawn toward grip bars attached to a force sensor (Shandong Academy of Medical Sciences, China), and then they were allowed to grab the bars with both front paws. The mice were slowly pulled straight back with consistent force until they released their grip. Grip strength was tested by the same investigator (Liu P.) three times to mitigate interrater differences in tensile strength, and the average value was used for comparative analyses.

### 2.6. Rotarod Test

The rotarod test was performed as described by van Dellen et al. [10] in mice from 8 to 20 weeks old. Two days before the test, mice were exposed to a training session to acclimatize them to the rotarod procedure. On the day of the test, three separate trials began at an initial rate of 3.5 rpm with an acceleration of 20 rpm/min to a maximum of 30 rpm over a period of 180 sec in the rotarod apparatus (Shanghai Xinruan, China). The latency to fall values were recorded, and the average time to fall was used in comparative analyses. After each test, the rods and separating walls were cleaned using ethanol (10%) to eliminate olfactory cues.

### 2.7. Novel Object Recognition Test

The novel object recognition test was performed at 20 weeks old, as described in the authors' previous report [11]. The apparatus consists of a square box (length 50 cm × width 50 cm × height 15 cm). On the first two days, the mice were habituated to the equipment for 10 min. On the test day, two identical objects, A1 and A2, were placed at the center of the box. The mouse was placed in the box and permitted to explore for 5 min. After a 1 h intertrial interval, the familiar object A2, was replaced with a novel object B, and the mouse was permitted to explore the objects for an additional 5 min, which was the 1 h retention trial. After a 24 h retention interval, object B was replaced with a novel object C, and the mouse was permitted to explore the objects for 5 min, which was a 24 h retention trial. The exploration time for each object was recorded. After each test, the floor was cleaned using ethanol (10%) to eliminate olfactory cues.

The preferential index (PI) was calculated as follows: time spent exploring the novel object/total exploration time.

### 2.8. Y Maze Test

The Y maze test was performed using 20-week-old mice, as described in the authors' previous report [11]. The apparatus comprised three brown wooden arms (length 40 cm × height 12 cm × width 10 cm). The mice were placed (individually) at the end of an arm and permitted to explore for 5 min. The total number of arm entries (*n*) and the sequence of entries were recorded. Once the mouse entered three arms continuously, it was defined a “successful alternation.” After each test, the floor was cleaned using ethanol (10%) to eliminate olfactory cues. Alternation behavior was calculated as follows: number of successive alternations/(*n* − 2) × 100.

### 2.9. Passive Avoidance Test

The passive avoidance test was performed using 20-week-old mice. The experimental device consisted of a bright and dark room. A powerful light bulb was hung at the top of the bright room to prompt the mice to enter into the dark room, the floor of which was equipped with an electrified copper plate that could deliver a small electric shock (31 V). The experiment was performed for two days. On the first day (training phase), the mice were placed individually into the bright room back to the hole without electricity and free for 3 min. The animals were then driven into the darkroom through the alternating current. The normal reactions of the mice were to run back to the bright room to avoid electric shock. Most of the animals, again, or repeatedly ran into the darkroom but were shocked and quickly ran back to the bright room. The number of times the mice entered into the darkroom again after a shock within 5 min were recorded as error times. The method was the same as the training phase day of the test phase, performed after a 24 h retention interval. After each test, the floor was cleaned using ethanol (10%) to eliminate olfactory cues.

### 2.10. Estimation of Oxidative Stress

Malondialdehyde (MDA), nitrite, superoxide dismutase (SOD), catalase (CAT), total glutathione, and oxidized glutathione levels were measured from brain extracts prepared in cell lysis buffer using commercially available assay kits (Nanjing Jiancheng Bioengineering Institute, Nanjing, China) according to the manufacturer's protocol. According to the kit instructions, the content of reduced gluthathione = total gluthathione − 2 × oxidized gluthathione.

### 2.11. Isolation of Total RNA and Reverse Transcriptase-Polymerase Chain Reaction (RT-PCR) Analysis

RT-PCR was performed according to the method described in the authors' previous report [12]. Cerebral cortex total RNA was extracted using TRIzol, and 2.0 *μ*g RNA was reverse transcribed by using a complementary DNA (cDNA) synthesis system. cDNA products were amplified using PCR with specific primers of glutathione peroxidase (GSH-Px) (forward (F): 5′-GGGACTACACCGAGATGAACG-3′; reverse (R): 5′-TCCGCAGGAAGGTAAAGAGC-3′); Nrf2 (F: 5′-CTTCCATTTACGGAGACCC-3′; R: 5′-GAGCACTGTGCCCTTGAGC-3′), and *β*-actin (F: 5′-CTGTGCCCATCTACGAGGGCTAT-3′; R: 5′-TTTGATGTCACGCACGATTTCC-3′). The amplified PCR products were separated on 1.5% agarose gels and then visualized with ethidium bromide under ultraviolet light. The band intensity was quantified using Gel-Pro-Analyzer software.

### 2.12. Immunohistochemical Staining

Immunohistochemical staining was performed in accordance with the method described in the authors' previous report [13]. Brain sections were incubated with EM48 (1 : 100) at 4°C overnight and then washed with phosphate-buffered saline (PBS) three times. The sections were incubated with biotin-labelled secondary antibody at 37°C for 30 min. The sections were treated with an avidin-biotin enzyme reagent and visualized using a DAB kit (Boster, Wuhan, China). The intensity and positive area of each section were quantified using ImageJ software.

### 2.13. Western Blotting Analysis

Western blotting analysis was performed in accordance with the method described in the authors' previous report [14]. Protein samples (30 *μ*g) were electrophoresed on an 8%–12% gradient sodium dodecyl polyacrylamide gel, and then they were transferred to PVDF membranes. The membranes were first blocked with 5% skim milk for 2 h at room temperature, incubated with primary antibodies EM48 (1 : 100), Sp1 (1 : 1000), LC3 (1 : 1000), P62 (1 : 1000), NQO1 (1 : 800), HO1 (1 : 800), and *β*-actin (1 : 1000) at 4°C overnight, then incubated with secondary antibody for 2 h at room temperature. Protein bands were visualized using a commercially available electrochemiluminescence kit. The band intensity was quantified using ImageJ software.

### 2.14. Cell Viability

PC12 cells were obtained from the National Infrastructure of Cell Line Resource (Beijing, China). The cells were cultured in RPMI-1640 medium containing 10% fetal bovine serum in a humidified cell incubator in an atmosphere of 5% CO_2_ at 37°C. The viability of cells was tested using the MTT assay. PC12 cells were plated in 96-well plates (1 × 10^4^ cells/well), and after 24 h of culture, the cells were treated with tolfenamic acid (5 and 10 *μ*M)/ML385 (5 *μ*M)/BSO (15 *μ*M) and cultured for an additional 24 h. Subsequently, the cells were incubated with 3-NP (15 mM) for 8 h, followed by addition of 5 mg/mL MTT to each well. After 4 h, the medium was removed and 150 *μ*L of dimethyl sulfoxide was added to each well. The absorbance was measured at a wavelength of 540 nm using an ELISA plate reader (Thermo Fisher Scientific, Waltham, MA, USA).

Control siRNA/Nrf2 siRNA (60 nM) were transfected into cells using Lipofectamine 2000 (Invitrogen, USA) according to the manufacturer's protocol. After 24 h of incubation, the cells were treated with tolfenamic acid (5 and 10 *μ*M) for an additional 24 h. Subsequently, the cells were incubated with 3-NP (15 mM) for 8 h, followed by cell viability analysis as described above.

The selection of tolfenamic acid/Nrf2 siRNA/ML385/BSO/3-NP doses and action times were based on the studies by Adwan et al., Huang et al., Zhang et al., Kulasekaran and Ganapasam, Jiang et al., and Speen et al. [15–20] and our preliminary experiments.

### 2.15. Measurement of Reactive Oxygen Species Levels

PC12 cells (1 × 10^4^ cells per well) were incubated with tolfenamic acid (5 and 10 *μ*M) for 24 h, after which 3-NP (15 mM) was added for an additional 8 h. After the drug treatments, the cells were incubated with 10 *μ*M DCF-DA, as the fluorescent probe, at 37°C for 30 min. The level of reactive oxygen species (ROS) was measured by using a commercially available assay kit (Beyotime Biotechnology, China) according to the manufacturer's instructions. Fluorescence was measured using a fluorometer (Thermo Fisher Scientific, USA) equipped with a 488 nm excitation filter and a 525 nm emission filter. DCF-DA is poorly selective for O_2_^•-^. Therefore, 50 *μ*M dihydroethidium (DHE) was also used as an O_2_^•-^ fluorescent probe. After 20 min incubation at 37°C, cells were examined under a fluorescence microscope (Olympus, Japan).

### 2.16. Statistical Analysis

The data were analyzed using SPSS version 21.0 (IBM Corporation, Armonk, NY, USA). The statistical significance of differences was determined using one-way ANOVA followed by Fisher's least significant difference multiple comparison test with homogeneity of variance or Dunnett's T3 test with heterogeneity of variance. Experimental data are expressed as mean ± SD or SEM; *p* < 0.05 was considered to be statistically significant. The power calculation was performed using G^∗^Power 3.1.9.2 (Heinrich-Heine-Universität Düsseldorf, Germany), and the value of 1 − *β* > 0.8 is acceptable.

## 3. Results

### 3.1. Effects of Tolfenamic Acid on Body and Brain Weight and Motor Deficits in R6/1 Mice

**Figure 1:**
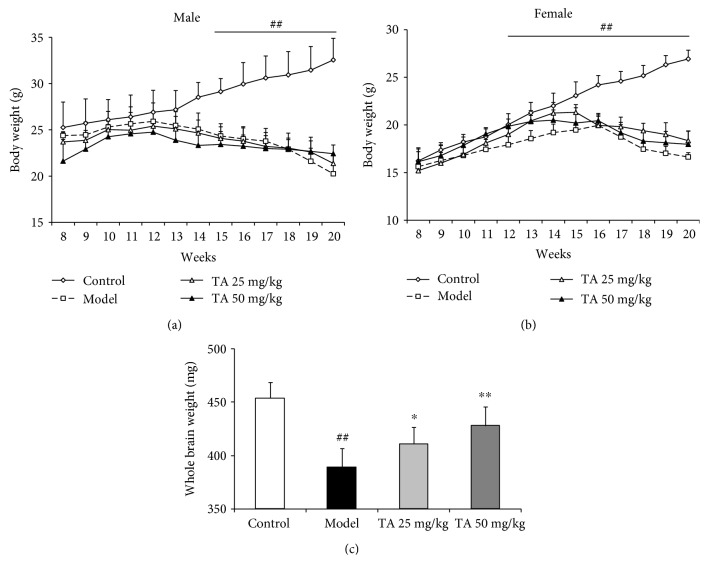
Effect of tolfenamic acid on body weight and brain weight in R6/1 mice. R6/1 mice exhibited a progressive decrease in body weight (a, b) and a decrease in brain weight (c) compared with control mice. Tolfenamic acid treatment attenuated losses in brain weight, but not body weight. All results are expressed as mean ± SD. *n* = 7–8; ^##^ *p* < 0.01 vs. control; ^∗^ *p* < 0.05 and ^∗∗^ *p* < 0.01 vs. model.

The body weight of the mice was tested as an index of general health, and brain weights were tested as an index of brain injury. Tolfenamic acid rescued changes in brain weight, but not body weight. The body weights of R6/1 mice progressively decreased from 15 to 20 weeks of age (*p* < 0.01) (Figures 1(a) and 1(b)); however, tolfenamic acid treatment did not result in any differences. At the beginning of the study, there were some differences in body weight between the male and female R6/1 mice; therefore, the data from male and female mice were analyzed individually. Compared with the control group mice, R6/1 group mice exhibited lighter brain weights (*F*(3, 21) = 20.789, *p* < 0.01; post hoc, *p* < 0.01) (Figure 1(c)). Tolfenamic acid treatment appeared to prevent a decrease in brain weight (*p* < 0.05) (Figure 1(c)).

**Figure 2:**
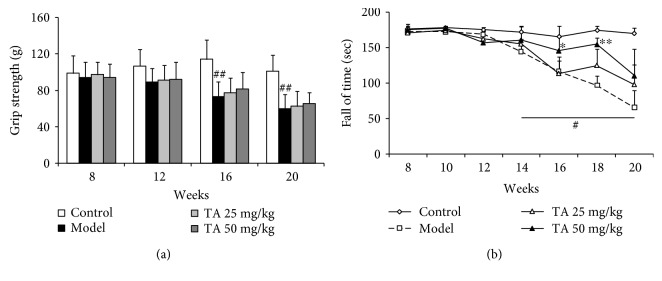
Effect of tolfenamic acid on motor deficits in R6/1 mice. R6/1 mice exhibited progressive weakening in muscle strength in the grip strength test (a) and a decrease in fall latency time (b) in the rotarod test compared with control mice. Tolfenamic acid treatment improved performance in the rotarod test, but not in the grip strength test. All results are expressed as mean ± SD. *n* = 7–8; ^#^ *p* < 0.05 and ^##^ *p* < 0.01 versus control; ^∗^ *p* < 0.05 and ^∗∗^ *p* < 0.01 versus model.

To assess muscle strength, a grip strength test was performed on the mice at 8, 12, 16, and 20 weeks of age. The grip strength of mice in the model group gradually decreased: 8 weeks, *F*(3, 33) = 0.181, *p* = 0.909; 12 weeks, *F*(3, 31) = 1.798, *p* = 0.168; 16 weeks, *F*(3, 28) = 8.487, *p* < 0.01, post hoc, *p* < 0.01; and 20 weeks, *F*(3, 26) = 12.041, *p* < 0.01, post hoc, *p* < 0.01 (Figure 2(a)). Tolfenamic acid treatment did not attenuate the weakening of muscle strength.

**Figure 3:**
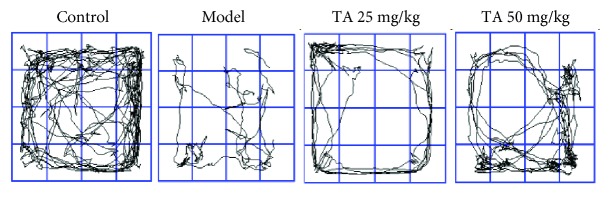
Representative trace plot for the locomotor behavior test.

The rotarod test and locomotor behavior test were performed in R6/1 mice to evaluate motor coordination. Post hoc analyses revealed that mice in the model group exhibited a short latency from 14 weeks and gradually decreased to 20 weeks (*p* < 0.05) (Figure 2(b)). Compared with the model group, tolfenamic acid (50 mg/kg) treatment significantly increased the latency to fall values at 16 and 18 weeks (*p* < 0.05) (Figure 2(b)). On the other hand, model group mice exhibited higher immobility time compared with control group mice (Figure 3), which reflected locomotor activity deficits in R6/1 mice (exploration distance: *F*(3, 21) = 25.899, *p* < 0.01, post hoc, *p* < 0.01; movement speed: *F*(3, 21) = 25.894, *p* < 0.01, post hoc, *p* < 0.01; exploration number: *F*(3, 21) = 2.218, *p* = 0.110; resting time: *F*(3, 21) = 16.495, *p* < 0.01, post hoc, *p* < 0.01; exploration time: *F*(3, 21) = 16.502, *p* < 0.01, post hoc, *p* < 0.01) (Table 1). Both doses of tolfenamic acid, however, rescued this change in locomotor activity (*p* < 0.05) (Table 1).

### 3.2. Effects of Tolfenamic Acid on Cognitive Dysfunction in R6/1 Mice

**Figure 4:**
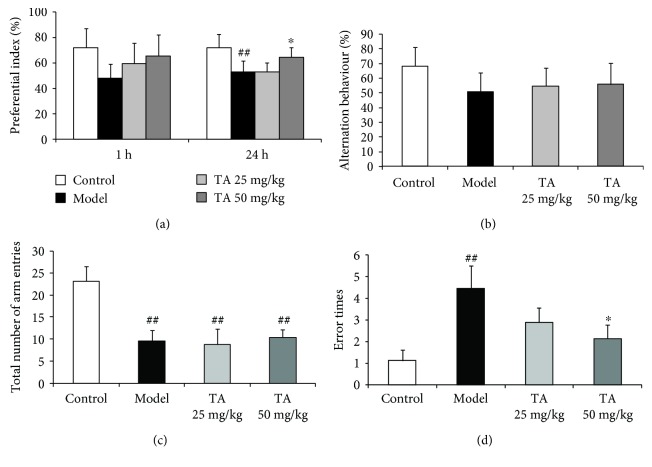
Effects of tolfenamic acid on cognitive dysfunction in R6/1 mice. R6/1 mice exhibited memory recall and visual recognition deficits in the novel object recognition test (a), a decrease in spontaneous alternation behavior and arm entries in the Y maze test (b, c), and more error times in the passive avoidance test (d) compared to control mice. Tolfenamic acid treatment attenuated the cognitive deficits in the novel object recognition test and the passive avoidance test, but not in the Y maze test. All results are expressed as the means ± SD. *n* = 7-8; ^##^ *p* < 0.01 versus control; ^∗^ *p* < 0.05 versus model.

The novel object recognition test was performed in mice individually to evaluate recall memory. Compared with the control group, the PI for novel object C was decreased in model group mice (*F*(3, 21) = 9.492, *p* < 0.01; post hoc, *p* < 0.01) (Figure 4(a)). More specifically, there were memory recall and visual recognition impairments in 20-week-old R6/1 mice. Treatment with 50 mg/kg tolfenamic acid significantly prevented the decrease in PI (*p* < 0.01) (Figure 4(a)). In the Y maze test, treatment with 25 or 50 mg/kg tolfenamic acid neither alleviated nor worsened spontaneous alternation behavior impairment in the mice (*F*(3, 21) = 2.510; *p* = 0.081) (Figure 4(b)). Furthermore, compared with the control group, model group and tolfenamic acid group mice exhibited less locomotor behavior during exploration in the Y maze test (total number of arm entries: *F*(3, 21) = 42.848, *p* < 0.01; post hoc, *p* < 0.01) (Figure 4(c)). In the passive avoidance test, compared with the control group, the error times in model group mice were significantly increased (*F*(3, 21) = 3.632, *p* < 0.05, post hoc, *p* < 0.01) (Figure 4(d)). The error times in the tolfenamic acid group (50 mg/kg) were less than that in R6/1 mice (*P* < 0.05, Figure 4(d)).

G^∗^Power software was used to perform the power calculation for all of the behavioral experiments. The data are provided as supporting information (SI.1-3). The value of 1 − *β* > 0.8 was acceptable.

### 3.3. Effect of Tolfenamic Acid on the Expression of mHtt and Autophagy Pathway in the Striatum of R6/1 Mice

**Figure 5:**
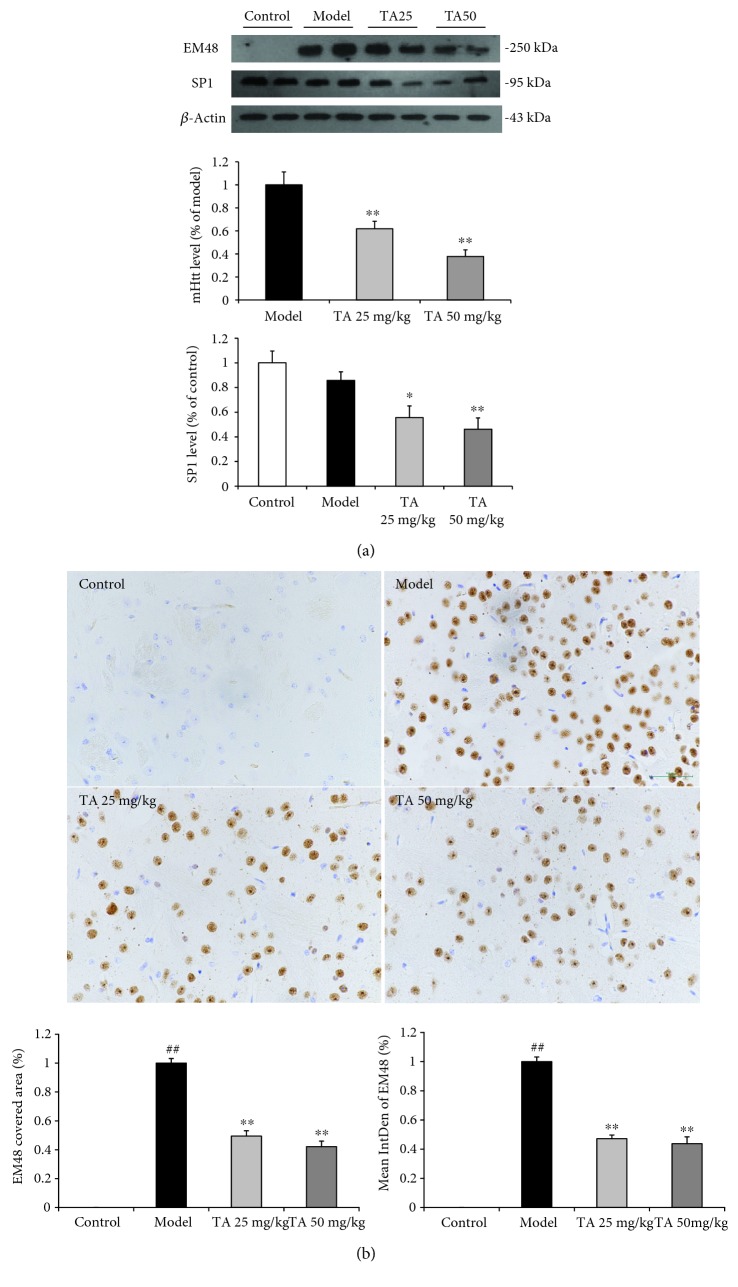
Effect of tolfenamic acid treatment on the expression of mutant huntingtin (Htt) and Sp1 in the striatum. Both doses of tolfenamic acid (25 or 50 mg/kg) significantly decreased the expression of mHtt and Sp1 (a) and decreased the intensity and positive area of the EM48-positive cell (b) in the striatum. All results are expressed as mean ± SEM. WB, *n* = 6; immunohistochemistry, *n* = 5, bar = 50 *μ*m. ^∗^ *p* < 0.05 and ^∗∗^ *p* < 0.01 vs. model.

**Figure 6:**
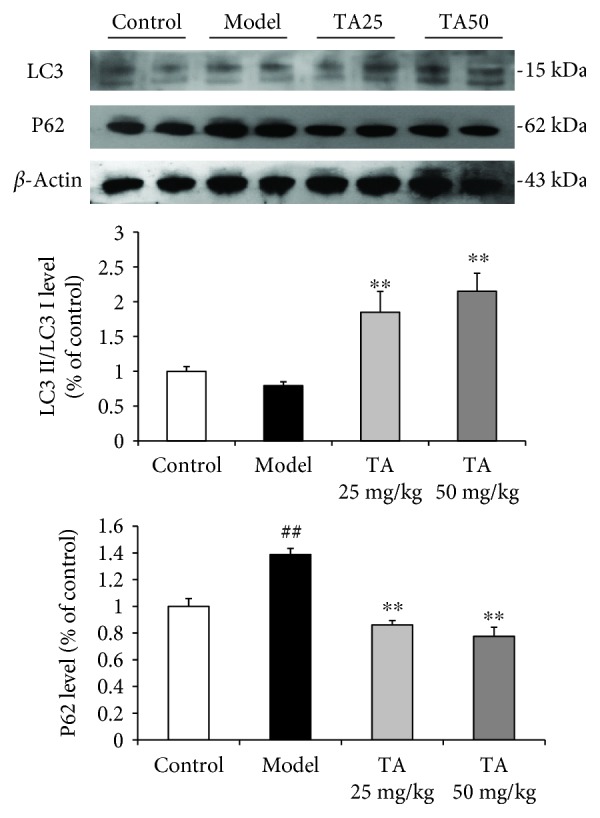
Effect of tolfenamic acid on the autophagy pathway in the striatum. Both doses of tolfenamic acid significantly increased the LC3-II/LC3-I ratio and decreased the expression of P62. All results are expressed as the mean ± SEM. *n* = 6; ^##^ *p* < 0.01 versus control; ^∗∗^ *p* < 0.01 versus model.

Western blotting and immunohistochemistry methods were used to examine the expression of mHtt in the striatum to investigate the neuroprotective effect of tolfenamic acid. Both doses of tolfenamic acid significantly decreased the expression of mHtt and Sp1 (mHtt: *F*(3, 15) = 14.545, *p* < 0.01; post hoc, *p* < 0.01; Sp1: *F*(3, 20) = 7.222, *p* < 0.01, post hoc, *p* < 0.05) (Figure 5(a)). In immunohistochemistry, R6/1 mice exhibited high levels of EM48 labeling in the striatum. EM48 labeling was reduced after TA treatment (positive area %: *F*(2, 12) = 76.893, *p* < 0.01, post hoc, *p* < 0.01; intensity %: *F*(2, 12) = 78.223, *p* < 0.01, post hoc, *p* < 0.05) (Figure 5(b)). However, the mechanism of how tolfenamic acid cleared misfolded proteins remained unclear and, accordingly, the autophagy pathway was investigated. Compared with the model group, the LC3-II/LC3-I ratio in the tolfenamic acid group was significantly increased (*F*(3, 20) = 10.665, *p* < 0.01, post hoc, *p* < 0.01) (Figure 6), suggesting that tolfenamic acid could increase autophagic function. P62 is an expendable substrate that decreases with autophagic upregulation. A decrease in P62 was also found after treatment with tolfenamic acid (*F*(3, 20) = 26.307, *p* < 0.01, post hoc, *p* < 0.01) (Figure 6).

### 3.4. Effect of Tolfenamic Acid on Oxidative Damage in R6/1 Mice

**Figure 7:**
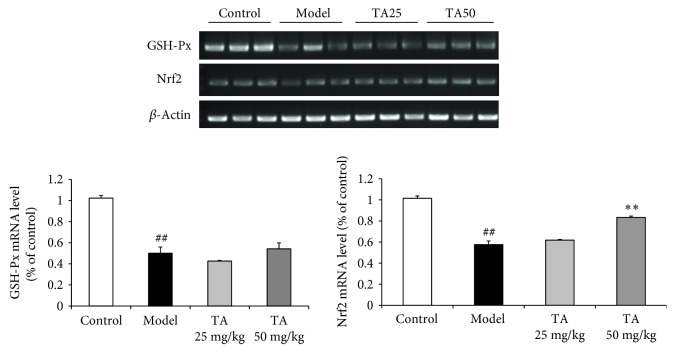
Effect of tolfenamic acid on messenger RNA (mRNA) levels of glutathione peroxidase (GSH-Px) and Nrf2 in the cortex. R6/1 mice exhibited oxidative damage in the cortex. Tolfenamic acid increased the mRNA level of Nrf2, but had no effect on GSH-Px. All results are expressed as mean ± SD. *n* = 3; ^##^ *p* < 0.01 versus control; ^∗∗^ *p* < 0.01 versus model.

**Figure 8:**
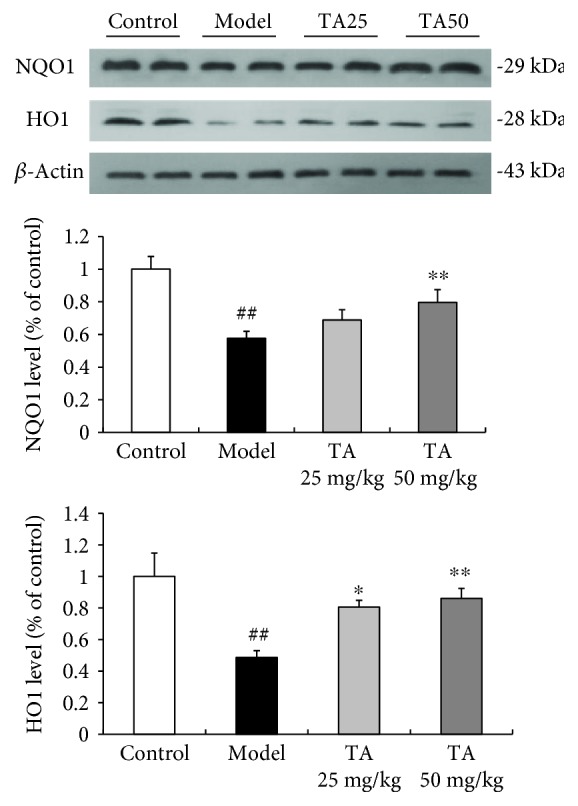
Effect of tolfenamic acid on the expression of NQO1 and HO1 in the cerebral cortex. Tolfenamic acid significantly increased the expression of NQO1 and HO1. All results are expressed as mean ± SEM. *n* = 6. ^##^ *p* < 0.01 versus control; ^∗^ *p* < 0.05 and ^∗∗^ *p* < 0.01 versus model.

Post hoc analyses revealed that there was no significant changes in the level of MDA, NO, CAT, or SOD among the groups (MDA: *F*(3, 16) = 2.357, *p* = 0.110; NO: *F*(3, 16) = 0.882, *p* = 0.471; CAT: *F*(3, 16)=0.922, *p* = 0.453; SOD: *F*(3, 16) = 2.020, *p* = 0.152) (Table 2), only the level of GSH decreased in R6/1 mice (total GSH: *F*(3, 16) = 2.357, *p* = 0.548; oxidized GSH: *F*(3, 16) = 14.380, *p* < 0.01, post hoc, *p* < 0.01; reduced GSH: *F*(3, 16) = 14.578, *p* < 0.01, post hoc, *p* < 0.01) (Table 3). Tolfenamic acid treatment significantly increased GSH levels in R6/1 mice (*p* < 0.01) (Table 3). To reconfirm this result, the gene expression level of GSH-Px in the cortex was tested using RT-PCR. TA neither decreased nor enhanced messenger RNA (mRNA) levels of GSH-Px at 20 weeks (*F*(3, 8) = 117.345, *p* < 0.01, post hoc, *p* = 0.330) (Figure 7(a)). TA increased the mRNA level of the antioxidant gene Nrf2 (*F*(3, 8) = 269.915, *p* < 0.01, post hoc, *p* < 0.01) (Figure 7). NQO1 and HO1 are two target genes of Nrf2. TA significantly increased the expression of NQO1 and HO1 in the cerebral cortex of R6/1 mice (NQO1: *F*(3, 20) = 6.338, *p* < 0.05, post hoc, *p* < 0.01; HO1: *F*(3, 20) = 7.296, *p* < 0.01, post hoc, *p* < 0.05) (Figure 8).

### 3.5. Effect of Tolfenamic Acid on 3-NP-Induced Neurotoxicity in PC12 Cells

**Figure 9:**
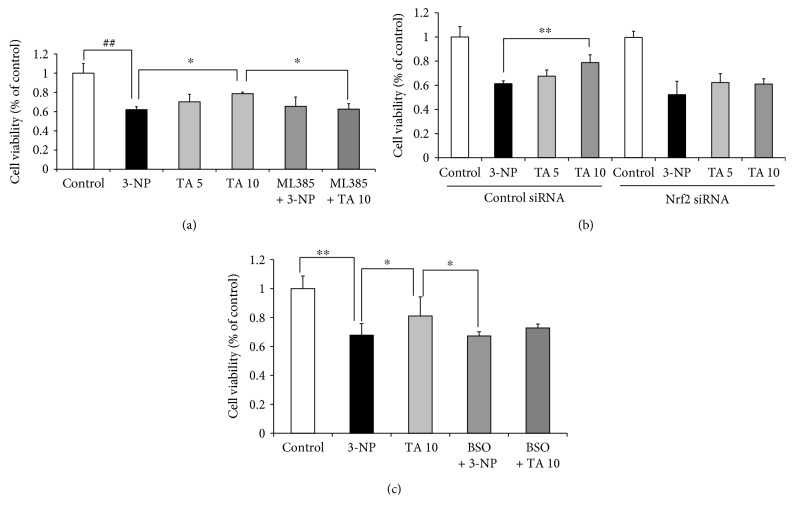
Effect of tolfenamic acid on the viability of PC12 cells cultured with 3-nitropropionic acid (3-NP). PC12 cells were preincubated with control small interfering RNA (siRNA) or Nrf2 siRNA (60 nM). After 24 h of incubation, the cells were treated with tolfenamic acid (5 and 10 *μ*M) for 24 h and then with 3-NP (15 mM) for 8 h (a). PC12 cells were treated with tolfenamic acid (5 and 10 *μ*M) with or without ML385 (5 *μ*M) for 24 h and then with 3-NP (15 mM) for 8 h (b). PC12 cells were treated with tolfenamic acid (10 *μ*M) with or without BSO (15 *μ*M) for 24 h and then with 3-NP (15 mM) for 8 h (c). After treatment, cell survival was determined using the MTT assay. All of the results are expressed as the mean ± SD. *n* = 3; ^∗∗^ *p* < 0.01 and ^∗^ *p* < 0.05.

Oxidative stress is the major cause of cellular injury in neurodegenerative disease. PC12 cells were incubated with 3-NP as the in vitro model to test the effect of tolfenamic acid on 3-NP-induced neurotoxicity and oxidative stress. The selection of tolfenamic acid/Nrf2 siRNA/ML385/BSO/3-NP doses and action times was based on the studies by Adwan et al., Huang et al., Zhang et al., Kulasekaran and Ganapasam, Jiang et al., and Speen et al. reported in [15–20] and our preliminary experiment. Pretreatment with tolfenamic acid for 24 h significantly prevented PC12 cell death caused by 3-NP exposure (*p* < 0.01) (Figures 9(a) and 9(b)). This protective effect was significantly blocked by Nrf2 siRNA and ML385 (a Nrf2 inhibitor) (Nrf2 siRNA: *F*(7, 16)=21.374, *p* < 0.01; post hoc, *p* < 0.01) (Figure 9(a)); (ML385: *F*(5, 12)=12.666, *p* < 0.01, post hoc, *p* < 0.05) (Figure 9(b)). BSO, a glutathione synthase inhibitor, could partly block the protective effect of tolfenamic acid, but the difference was not statistically significant (*F*(4, 10) = 7.695, *p* < 0.01, post hoc, *p* = 0.263) (Figure 9(c)). These results suggest that tolfenamic acid exerted its neuroprotective effect through its antioxidant properties.

### 3.6. Effect of Tolfenamic Acid on 3-NP-Induced ROS Generation in PC12 Cells

**Figure 10:**
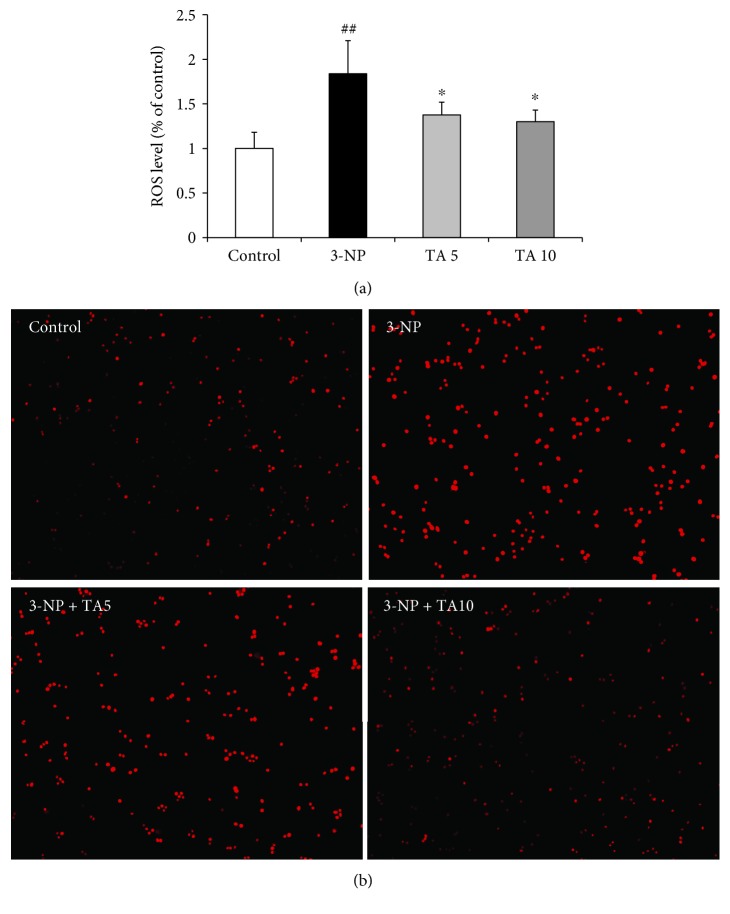
Effect of tolfenamic acid on reactive oxygen species production in PC12 cells cultured with 3-nitropropionic acid (3-NP). PC12 cells were treated with tolfenamic acid (5 and 10 *μ*M) for 24 h and then with 3-NP (15 mM) for 8 h. DCF-DA (a) and DHE (b) were used as the fluorescent probe. All results are expressed as $\text{mean}\pm \text{S}\underset{¯}{\text{D}}$. *n* = 3; ^##^ *p* < 0.01 versus control; ^∗^ *p* < 0.05 versus 3-NP. Magnification ×10.

Compared with the control group, 3-NP treatment significantly increased ROS (DCF) production in PC12 cells (*F*(3, 8) = 6.949, *p* < 0.05, post hoc, *p* < 0.01) (Figure 10). Tolfenamic acid protected PC12 cells by decreasing ROS accumulation (*p* < 0.05) (Figure 10). DHE was used to reflect O_2_^•-^ accumulation. The red fluorescence in PC12 cells indicated that 3-NP caused the generation of O_2_^•-^. Tolfenamic acid decreased O_2_^•-^ levels in 3-NP-treated PC12 cells. These results reconfirmed the antioxidant effect of tolfenamic acid.

## 4. Discussion

NSAIDs have been reported to alter HD pathology and attenuate motor deficits in HD animal models through mechanisms of cyclooxygenase inhibition, and they have led researchers to consider NSAIDs as potential anti-HD agents [22, 23]. The expression of Sp1 is elevated in the brains of transgenic mouse models of HD and HD patients [24–26]. SP1 regulates the transcription of Htt, and many target genes of Sp1 have been reported to be upregulated in HD [3, 5]. Mutant Htt-induced oxidative stress can activate Sp1 in neurons and glial cells [21]. Activated Sp1 further exacerbates neuroinflammatory reaction and oxidative stress [27, 28]. Sp1 knockout HD transgenic mice live longer than their HD counterparts [25]. These data suggest that the upregulation of Sp1 contributes to the pathology of HD, and that suppression of Sp1 may be beneficial.

Tolfenamic acid can induce the proteasome-dependent degradation of SP transcription factors [6]. Previous studies have demonstrated that Sp1 overexpression upregulates APP and BACE1 expression, which is involved in Alzheimer's disease [7]. Tolfenamic acid can attenuate cognitive deficits in APP transgenic mice after 2 weeks of administration [7, 29]. These positive effects in cognitive behavior are regulated by inhibiting Sp1 and its target genes *APP* and *BACE1*. Thus, we hypothesized that tolfenamic acid could inhibit Htt and, furthermore, attenuate motor and cognitive deficits in HD mice.

R6/1 mice exhibit progressive locomotor coordination deficits, which begin at approximately 3 months of age [10, 30]. In this study, motor impairment was assessed using the rotarod test. We found that tolfenamic acid could inhibit the progressive impairment of locomotor coordination. R6/1 mice also exhibited muscular weakness in the forelimb on the grip strength test and substantial locomotor activity decrease in the locomotor behavior test. However, tolfenamic acid partly mitigated these impairments in R6/1 mice. Cognitive deficits appear before motor deficits in patients with HD [31]. HD patients experience a more serious decline of memory recall function than memory storage, which is caused by neuronal and synaptic loss [32]. We used the novel object recognition test to evaluate the effect of tolfenamic acid on recall memory [33]. We also used the Y maze and passive avoidance tests to access the effect of TA on working and long-term memory. R6/1 mice exhibited significant learning and memory deficits, and tolfenamic acid increased PI in the novel object recognition test and decreased the error times in the passive avoidance test. Western blotting results revealed that tolfenamic acid reduced Htt aggregation in the striatum. Tolfenamic acid inhibited the expression of Sp1, which perhaps suggests that tolfenamic acid decreased the transcriptional level of mutant Htt in the brains of R6/1 mice. Activating autophagic function also contributed to the clearance of mutant Htt. LC3 transforms from form I to form II to serve as the recruiter of the autophagosome substrate P62 during the activation of autophagy. SP1 can block autophagic flux via activating P62, and Sp1 inhibition will promote autophagy [34, 35]. In this study, we found that tolfenamic acid significantly increased the LC3-II/LC3-I ratio and decreased the level of P62. Therefore, tolfenamic acid inhibits the transcription factor Sp1 and activates the autophagy pathway, which may contribute to the clearance of mutant Htt aggregates.

Another potentially important function of tolfenamic acid is the reduction of oxidative stress in the brain. mHtt causes inflammation, oxidative stress, lipid peroxidation, and mitochondrial dysfunction [36–38]. Oxidative stress can cause cellular damage and neurodegeneration by inducing the production of ROS. Nrf2 regulates antioxidant gene expression in response to oxidative stress [39]. We found that tolfenamic acid treatment significantly increased mRNA levels of Nrf2. NQO1 and HO1 are two vital target genes of Nrf2. We found that TA significantly increased the expression of NQO1 and HO1 in the cerebral cortex of R6/1 mice. Previous studies have reported that oxidative stress caused by elevated levels of free radicals and depleted antioxidant enzymes cause neuronal damage in HD animal model brains [40, 41]. However, in this study, compared with WT mice, the content of MDA, NO, CAT, and SOD did not change significantly—only the level of GSH decreased in R6/1 mice. Tolfenamic acid treatment significantly attenuated the GSH level in R6/1 mice. Previous studies have reported that the level of GSH is decreased in HD patients [42]. GSH is produced in the cytosol and transferred to the nuclei or mitochondria. When GSH is oxidized, it becomes oxidized GSH. However, in vitro, we found that compared with the tolfenamic acid treatment, the GSH synthase inhibitor BSO did not significantly block the protective effect of tolfenamic acid in PC12 cells. Therefore, regulating the stabilization of GSH and oxidized GSH may be only one mechanism for tolfenamic acid to cure HD, and will be investigated in future studies. Kulasekaran and Ganapasam reported that 3-NP caused PC12 cell injury and induced significantly elevated ROS production [18]. Therefore, we used this in vitro cell model to reconfirm the antioxidant effect of tolfenamic acid. Tolfenamic acid significantly prevented 3-NP-induced neurotoxicity in PC12 cells, and this effect could be partly inhibited by Nrf2 siRNA or the specific Nrf2 inhibitor—ML385. Tolfenamic acid also decreased ROS production in PC12 cells.

## 5. Conclusions

Collectively, the results of the present study suggest that tolfenamic acid can attenuate motor and cognitive deficits in R6/1 transgenic mice. Tolfenamic acid could promote the degradation of mHtt by inhibiting the transcription factor Sp1 and enhancing autophagic function. Antioxidant production in the brains of R6/1 mice and in PC12 cells is another important mechanism of tolfenamic acid. It has been established that tolfenamic acid is safe for clinical use. Therefore, our data support tolfenamic acid as a potential candidate for the treatment of HD.

## Figures and Tables

**Table 1:** Effect of tolfenamic acid on locomotor behavior in R6/1 mice.

Group	Exploration distance (mm)	Movement speed (mm/s)	Exploration number	Resting time (s)	Exploration time (s)
Control	13,550.80 ± 956.50	45.17 ± 3.19	37.25 ± 2.86	32.43 ± 5.25	267.57 ± 5.25
Model	2699.30 ± 674.67^a^	9.01 ± 2.25^a^	33.14 ± 4.19	187.72 ± 22.06^a^	111.87 ± 22.19^a^
TA 25 mg/kg	6000.28 ± 757.01^b^	20.00 ± 2.52^b^	46.88 ± 4.36	130.22 ± 18.48^b^	169.79 ± 18.48^b^
TA 50 mg/kg	7674.76 ± 1149.21^c^	25.58 ± 3.83^c^	39.57 ± 4.09	72.19 ± 17.46^c^	227.81 ± 17.46^c^

All of the results are expressed as the means ± SEM. *n* = 7 or 8. ^a^ *p* < 0.01 vs. the control group; ^b^ *p* < 0.05 and ^c^ *p* < 0.01 vs. the model group.

**Table 2:** Effect of tolfenamic acid on oxidative damage (lipid peroxidation, nitrite, superoxide dismutase, and catalase levels) in the brain of R6/1 mice.

Group	MDA (nmol/mg protein)	NO (*μ*mol/mg protein)	SOD (U/mg protein)	CAT (U/mg protein)
Control	9.10 ± 1.16	157.81 ± 17.67	27.33 ± 5.71	16.14 ± 2.57
Model	12.23 ± 2.38	183.516 ± 34.48	23.52 ± 3.06	12.69 ± 5.26
TA 25 mg/kg	10.88 ± 1.57	181.20 ± 30.14	23.19 ± 3.69	12.97 ± 3.72
TA 50 mg/kg	9.80 ± 2.52	171.202 ± 26.43	27.82 ± 1.91	14.34 ± 2.45

All of the results are expressed as the means ± SD. *n* = 5.

**Table 3:** Effect of tolfenamic acid on oxidative damage (glutathione levels) in the brain of R6/1 mice.

Group	Total glutathione (*μ*mol/L)	Oxidized glutathione (*μ*mol/L)	Reduced glutathione (*μ*mol/L)
Control	195.18 ± 26.99	51.05 ± 12.06	93.08 ± 13.99
Model	214.23 ± 27.76	95.20 ± 12.21^a^	23.83 ± 15.14^a^
TA 25 mg/kg	205.97 ± 9.01	70.50 ± 9.74^b^	64.97 ± 18.82^b^
TA 50 mg/kg	201.94 ± 12.41	66.97 ± 8.62^b^	68.00 ± 18.67^b^

All of the results are expressed as the means ± SD. *n* = 5. ^a^ *p* < 0.01 vs. the control group; ^b^ *p* < 0.01 vs. the model group.

## Data Availability

The data used to support the findings of this study are available from the corresponding author upon request.

## Abbreviations

CAT: Catalase
GSH-Px: Glutathione peroxidase
HD: Huntington's disease
HO1: Heme oxygenase 1
MDA: Malondialdehyde
mHtt: Mutant huntingtin protein
NO: Nitrite
NQO1: NAD(P)H quinine oxidoreductase 1
Nrf2: Nuclear factor erythroid 2-related factor 2
ROS: Reactive oxidative species
SOD: Superoxide dismutase
Sp1: Specificity protein 1
TA: Tolfenamic acid.

## Supplementary Materials

Supplementary Materials SI.1: the power (1 − *β*) value for the grip strength test and rotarod test. SI.2: the power (1 − *β*) value for the locomotor behavior test. SI.3: the power (1 − *β*) value for the passive avoidance test, Y maze test, and novel object recognition test.
