# Supplementary material for: Prevention of Huntington's Disease-Like Behavioral Deficits in R6/1 Mouse by Tolfenamic Acid Is Associated with Decreases in Mutant Huntingtin and Oxidative Stress
Source: Oxid Med Cell Longev. 2019 Mar 26;2019:4032428. doi: 10.1155/2019/4032428 (PMC6458866; doi:10.1155/2019/4032428)
Supplement: Supplementary Materials — SI.1: the power (1 − β) value for the grip strength test and rotarod test. SI.2: the power (1 − β) value for the locomotor behavior test. SI.3: the power (1 − β) value for the passive avoidance test, Y maze test, and novel object recognition test. [file 4032428.f1.doc]

Supporting information

SI.1 The Power (1-β) value for Grip strength test and Rotarod test

|  | Grip strength test | | | |  | Rotarod test | | | | | | |
| --- | --- | --- | --- | --- | --- | --- | --- | --- | --- | --- | --- | --- |
|  | 8 weeks | 12 weeks | 14 weeks | 20 weeks |  | 8 weeks | 10 weeks | 12 weeks | 14 weeks | 16 weeks | 18 weeks | 20 weeks |
| Power (1-β) | 0.071 | 0.374 | 0.936 | 0.993 |  | 0.999 | 0.999 | 1 | 0.999 | 0.999 | 1 | 1 |

SI.2 The Power (1-β) value for Locomotor behaviour test

|  | Locomotor behaviour test | | | | |
| --- | --- | --- | --- | --- | --- |
|  | Exploration  Distance | Movement Speed | Exploration number | Resting time | Exploration time |
| Power (1-β) | 0.999 | 0.999 | 0.758 | 1 | 1 |

SI.3 The Power (1-β) value for Passive avoidance test, Y maze test and Novel object recognition test

|  | Passive avoidance test |  | Y maze test | |  | Novel object recognition test | |
| --- | --- | --- | --- | --- | --- | --- | --- |
|  |  |  | Alternation behavior | Total number of arm entries |  | Preferential index 1h | Preferential index 24h |
| Power (1-β) | 0.975 |  | 0.571 | 1 |  | 0.692 | 0.931 |
